# Vanadium Compounds with Antidiabetic Potential

**DOI:** 10.3390/ijms242115675

**Published:** 2023-10-27

**Authors:** Luísa M. P. F. Amaral, Tânia Moniz, André M. N. Silva, Maria Rangel

**Affiliations:** 1 LAQV, REQUIMTE, Departamento de Química e Bioquímica, Faculdade de Ciências, Universidade do Porto, Rua do Campo Alegre, s/n, 40169-007 Porto, Portugal; luisaamaral@fc.up.pt (L.M.P.F.A.); taabreu@icbas.up.pt (T.M.); 2 LAQV, REQUIMTE, Instituto de Ciências Biomédicas de Abel Salazar, Universidade do Porto, Rua de Jorge Viterbo Ferreira 228, 4050-313 Porto, Portugal

**Keywords:** antidiabetic drugs, chemical speciation, insulin signaling, diabetes mellitus, metallopharmaceuticals, vanadium, vanadium complexes

## Abstract

Over the last four decades, vanadium compounds have been extensively studied as potential antidiabetic drugs. With the present review, we aim at presenting a general overview of the most promising compounds and the main results obtained with in vivo studies, reported from 1899–2023. The chemistry of vanadium is explored, discussing the importance of the structure and biochemistry of vanadate and the impact of its similarity with phosphate on the antidiabetic effect. The spectroscopic characterization of vanadium compounds is discussed, particularly magnetic resonance methodologies, emphasizing its relevance for understanding species activity, speciation, and interaction with biological membranes. Finally, the most relevant studies regarding the use of vanadium compounds to treat diabetes are summarized, considering both animal models and human clinical trials. An overview of the main hypotheses explaining the biological activity of these compounds is presented, particularly the most accepted pathway involving vanadium interaction with phosphatase and kinase enzymes involved in the insulin signaling cascade. From our point of view, the major discoveries regarding the pharmacological action of this family of compounds are not yet fully understood. Thus, we still believe that vanadium presents the potential to help in metabolic control and the clinical management of diabetes, either as an insulin-like drug or as an insulin adjuvant. We look forward to the next forty years of research in this field, aiming to discover a vanadium compound with the desired therapeutic properties.

## 1. Introduction

Discovered in the early XIX century [1], vanadium (V) has garnered significant interest from chemists, geologists, biologists, and biochemists, amongst others [2]. Ranking as the 5th most abundant transition metal in the Earth’s crust, it constitutes approximately 0.014% of the crust’s abundance. Despite its relatively low occurrence, vanadium exhibits widespread distribution and can vary considerably in concentration, with some deposits and freshwater sources containing notably high levels.

Since its discovery, vanadium has played a vital role in metallurgy, with yearly production surpassing 100,000 tons [3]. Its primary application lies in alloy production, particularly as an additive in steel manufacturing. Notably, there is currently no viable substitute for vanadium in aerospace titanium alloys, cementing its importance in this industry. Moreover, its economic significance has been on the rise, attributed to its usage in the emerging field of new-generation vanadium redox flow batteries [4]. Beyond metallurgy, vanadium has proven highly relevant in various scientific research domains, particularly in biomedical and health sciences, where its unique properties are being explored for potential medical applications [5,6,7].

Being ubiquitous in the Earth’s crust, vanadium accumulates and serves diverse functions in organisms such as bacteria, algae, fungi, plantae, and animals [8]. Various enzymes, such as bromoperoxidases in algae, haloperoxidases in macro-algae, nitrogenases in nitrogen-fixing bacteria, and chloroperoxidases in certain fungi, depend on vanadium for their correct functioning [9]. Despite the absence of a specific identified biological role for vanadium, it has also been shown that its deficiency poses a problem in birds, rodents, fish, and lower animals [10].

The essentiality of vanadium in human physiology has been a subject of great debate but remains largely unproven [1,10]. In humans, vanadium deficiency has been reported, while its acute and chronic toxicity has been also extensively documented. The awareness of vanadium’s physiological effects dates to the 1960s [11,12], and although the specific mechanisms mediating its physiological functions remain unknown, researchers have explored its potential application as a source for antitumor, anti-HIV, antituberculosis, and particularly as antidiabetic therapeutics [6]. Herein, we provide an overview of the most relevant studies on antidiabetic action reported from 1899–2023.

## 2. The Aqueous Chemistry of Vanadium

Vanadium is the third element of the first row of the transition metals and exhibits typical characteristics of early transition metals. It shows a preference for high oxidation states, high coordination numbers, and bonding with “hard”, negatively charged ligands, such as oxygen. In aqueous solutions, the most common oxidation states are +3, +4, and +5, with V(IV) and V(V) being prevalent in biological systems. These oxidation states tend to form oxides represented by the vanadate anion (VO_4_^3−^) and the vanadyl cation (VO^2+^). Notably, in the biological milieu, VO_4_^3−^ predominates in the oxidizing environment of the serum and extracellular fluid, while VO^2+^ appears to be more prevalent in the reducing intracellular environment [13]. At physiological pH values, vanadate exists as an equilibrium between H_2_VO_4_^−^ and HVO_4_^2−^ (pK_a_ = 7.8) [14].

Regarding the latter species, it is important to mention that as the solution becomes acidic H_2_VO_4_^−^ may undergo further protonation reactions that give rise to a different species, usually represented as the ion VO_2_^+^(aq) = [VO_2_(H_2_O)_4_]^−^, in which the geometry of the vanadium center changes from tetrahedral to pseudo-octahedral. The process is dependent on the pH and vanadium concentration. The protonation constants and redox behavior of this vanadate cation have been thoroughly studied by potentiometry and ^51^V NMR by Peterson et al. [15]. In acidic and reducing conditions the VO_2_^+^(aq) ion may undergo reduction originating the vanadyl ion, VO^2+^(aq). The equilibrium between the two vanadium cations is also well-established and characterized [15].

The latter equilibrium is particularly important in the discussion of the solution chemistry of vanadium complexes since both cations may undergo hydrolysis and ligand exchange giving rise to a variety of oxovanadium complexes with different coordination numbers, geometry, and nuclearity [16,17].

The biological activity of vanadium is thought to arise from the structural and electronic resemblance between VO_4_^3−^ and phosphate (PO_4_^3−^) [2,6,9,10]. Both species form tri-anions with a tetrahedral structure, contributing to their functional similarities. VO_4_^3−^ has been identified as an inhibitor of phosphatases, ATPases, and phosphorylases, suggesting its regulatory role in cellular processes. However, a notable distinction arises in the behavior of vanadate at neutral pH values, where it tends to undergo hydrolysis [16].

Inside cells, the vanadyl ion (VO^2+^) is usually predominant and exists associated with proteins or in its hydrated form ([VO(H_2_O)_5_]^2+^) [13]. At pH values lower than 3, [VO(H_2_O)_5_]^2+^ is stable, while hydroxy-vanadyl species emerge at pH levels above 4 [9]. At neutral pH, the aqueous chemistry is dominated by hydrolysis reaction and the solubility product of hydrolyzed species [VO(HO)_2_]_n_ [9,16]. However, at physiological concentrations (~30 nM), precipitation is not the prevalent outcome. Instead, both monomeric and dimeric anionic species, namely [VO(OH)_3_]^−^ and [(VO)_2_(OH)_5_]^−^, coexist, with the latter being more prevalent [9]. At neutral pH, VO^2+^ tends to undergo oxidation, potentially leading to the formation of VO_4_^3−^. It is still not clear whether it is the action of the negatively charged [VO(OH)_3_]^−^ or this propensity for oxidation that explains the ability of vanadyl to inhibit intracellular phosphatases.

Various spectroscopic techniques have been employed for the structural characterization and speciation of vanadium compounds. A comprehensive review of these methodologies can be found in the work of Pessoa et al. [17]. Magnetic resonance spectroscopy, including both Nuclear Magnetic Resonance (NMR) and Electron Paramagnetic Resonance (EPR), has been particularly instrumental in this field [9,17].

V(V) is a diamagnetic species with an electronic configuration of [Ar] 3d^0^, making it suitable for NMR studies. This can be achieved through more common ^13^C and ^1^H NMR studies of compounds directly interacting with the vanadium ion, or alternatively, by directly assessing ^51^V [2]. ^51^V possesses a nuclear spin of 7/2 and a natural abundance of 99.76%, making it an excellent NMR probe. Additionally, the chemical shift of ^51^V can be notably influenced by the coordination sphere around the vanadium atom.

V(IV) is a one-electron radical, characterized by an electron configuration of [Ar] 3d^1^, and its presence can be observed using EPR techniques. Notably, at room temperature, vanadium exhibits a distinctive 8-line EPR spectrum resulting from the 7/2 nuclear spin of ^51^V [9].

EPR and ^51^V NMR are not only useful to structurally characterize V(IV) and V(V) species per se but also to characterize oxidation-reduction processes undergone by vanadium complexes and the study of their interactions with biological membranes.

In order to illustrate the power of using both magnetic resonance techniques in tandem, we will focus on studies performed in our group regarding oxovanadium(IV) complexes of 3-hydroxy-4-pyridinone ligands [18,19,20,21].

**Figure 1:**
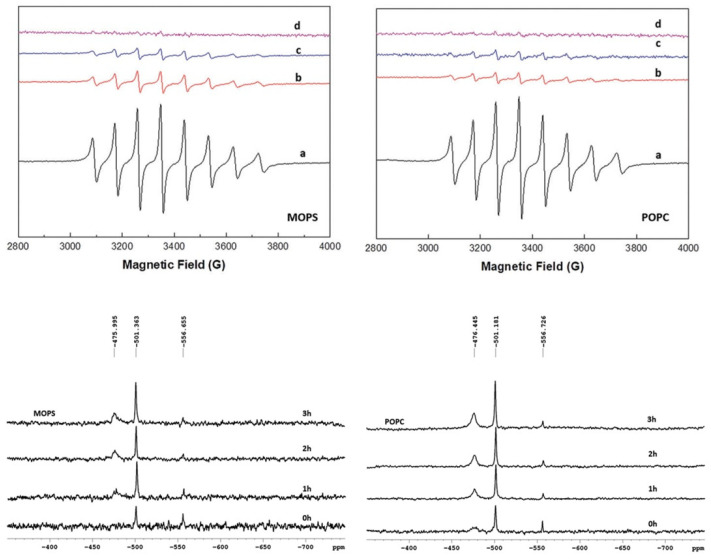
EPR spectra of VO(dmpp)_2_ in buffer (MOPS) (**upper panel**, **left**) and in liposome suspension (POPC) (**upper panel**, **right**) at 0 h (a); 1 h (b); 2 h (c) 3 h (d); ^51^V NMR spectra of VO(dmpp)_2_ in MOPS (**lower panel**, **left**) and POPC (**lower panel**, **right**) at 0 h, 1 h, 2 h and 3 h. Adapted and reproduced from [21].

**Figure 2:**
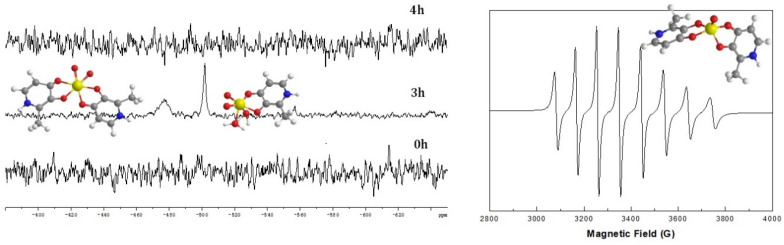
^51^V NMR for the complex VO(dmpp)_2_ in buffer at 0 h and 3 h and after the addition of sodium ascorbate (4 h) (**left**). EPR spectra of the complex in buffer (**right**) after the addition of sodium ascorbate (4 h). Adapted and reproduced from [21].

**Figure 3:**
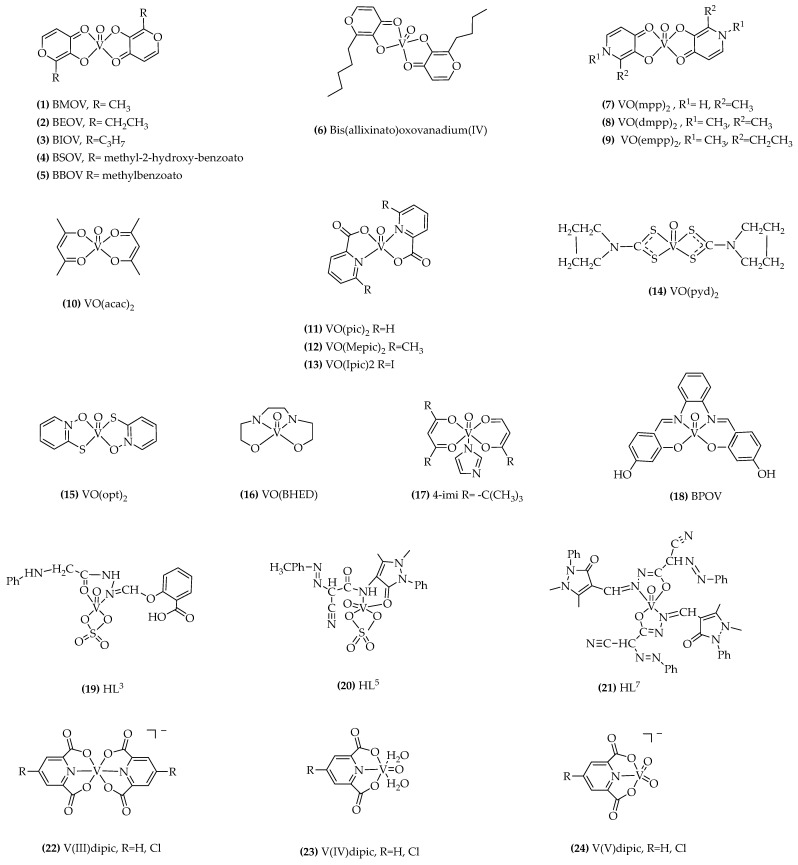
Representative formulae of the most relevant vanadium compounds tested in animal and human studies.

In the most recent study [21] we reported EPR and ^51^V NMR characterization of vanadium(IV/V) species, (Figure 1 and Figure 2), originating from bis(3-hydroxy-4-pyridinonato)oxidovanadium(IV) complexes, including VO(dmpp)_2_, ((**8**), Figure 3) in aqueous solution at pH 7.4 (MOPS buffer) under aerobic conditions and in liposome suspensions (POPC), in order to improve solubility and also foreseeing the potential of these systems as delivery vehicles.

Analysis of the EPR spectra of bis(3-hydroxy-4-pyridinonato)oxidovanadium(IV)complexes shows that upon dissolution a single species is present in solution, [VOL_2_], and that the use of liposome suspensions significantly improves solubility. In the presence of air [VOL_2_] is oxidized to three species, [VO_2_L_2_]^−^, [VO_2_L], and V1(H_2_VO_4_^−^) as characterized by the ^51^V NMR spectra. Also, we studied the systems for three hours following the oxidation process by monitoring both the EPR and NMR spectra of the solutions.

To mimic the potential effect of reducing ligands, present in the cell milieu, on the vanadium (V) species present in solution, we studied the effect of the addition of sodium ascorbate, and we verified that the latter is reduced to the original [VOL_2_] complex (Figure 2).

## 3. Antidiabetic Properties of Vanadium

Diabetes mellitus (DM) is classified as a pandemic by the World Health Organization (WHO), and it stands as a major risk factor contributing to the rising mortality rates from non-communicable diseases [22]. The hallmark of diabetes is hyperglycemia, a condition characterized by elevated blood sugar levels. The two main types of this disease arise from either lack or decreased insulin production, known as type 1 diabetes (T1D), or increased resistance to this hormone action, designated as type 2 diabetes [23]. In T1D, the insufficient production of insulin mostly results from β-cell death. Conversely, type 2 diabetes (T2D) arises from increased tissue resistance to insulin action. A significant number of patients ultimately require insulin therapy for the effective management of their condition. However, the increased resistance to insulin therapy among patients is a pressing issue for clinicians.

Until recently, insulin was the only pharmacotherapeutic option for the treatment of T1D, and a variety of insulin formulations (basal and prandial) and modes of administration (syringe, pen, prefilled pen, and pump) are available [24]. Alternative or adjuvant therapies include Pramlintide, an injectable amylin analog, and Teplizumab, a monoclonal antibody that specifically targets immune cells involved in the destruction of β-cells [25,26]. Therapies for T2D patients include several oral agents, with alpha-glucosidase inhibitors, metformin, dopamine-2 agonists, DPP-4 inhibitors, GLP-1 receptor agonists, meglitinides, Sodium-glucose transporter (SGLT) 2 inhibitors, sulfonylureas, and thiazolidinediones being the primary medications used to control diabetes [27,28,29].

The achievement of glycemic control through these treatments has been proven to reduce the complications associated with both types of diabetes. However, the physiology of glucose homeostasis is complex, and the use of insulin and oral agents corrects only part of the underlying pathophysiology of diabetes.

In this context, vanadium has garnered attention as a potential adjuvant therapy for diabetes due to its lack of deleterious effects on normal metabolism. vanadium is ubiquitous and naturally occurring in our organism [10]. It can be absorbed from dietary intake, drinking water, and even inhaled air through the lungs. Once absorbed, vanadium accumulates in most human organs, with approximately 50% being stored in the bones, while substantial reserves can also be found in the liver and spleen [10]. The clearance and residence times of vanadium vary significantly between organs, contributing to its distribution throughout the body, but most of the absorbed vanadium is excreted through the urine or feces, and at homeostatic levels, no toxicity has been reported [1,5].

The effects of vanadium on human metabolism have been recognized almost since its discovery. vanadium was observed to impact cholesterol production, leading to a reduction in blood plasma levels [30,31]. Further, it was described that it affects energy metabolism by inhibiting liver ATP production. A significant breakthrough occurred when vanadium was found to act as an inhibitor of membrane-bound Na^+^-K^+^ ATPase activity [32], prompting new research in the field. In 1979, Tollman demonstrated in a series of in vitro systems that vanadium affects glucose metabolism [33]. This was swiftly followed by Dubyak and Kleinzeller’s work showing stimulation of glucose oxidation by vanadyl and vanadate in rat adipocytes [34]. Finally, the seminal work by Heyliger and co-workers showed that sodium vanadate was effective at controlling hyperglycemia in vivo, using the streptozotocin (STZ)-induced diabetes rat model [35].

The described results led to a significant interest in the insulin-mimetic or insulin-like activity of vanadium. Several studies, reviewed below, were carried out initially with vanadium salts and ultimately with vanadium chelates. Vanadium administration was found to alleviate several diabetes-related metabolic changes while offering two advantages over insulin: it is orally active and seems to avoid the risk of hypoglycemia. Nevertheless, vanadium does not fully substitute insulin in any in vivo model of diabetes, and it is better described as having an insulin-enhancing effect.

When considering vanadium compounds as therapeutic agents, it must be highlighted that vanadium is known to be toxic [10,36]. V_2_O_5_ is a well-recognized environmental and occupational hazard, being a common source of pulmonary intoxication and ultimately pulmonary fibrosis [5]. Because vanadium accumulates in several organs and promotes oxidative stress, it has been described to have hepatotoxic, nephrotoxic, cardiotoxic, and neurotoxic actions [10]. Although disputed, vanadium is also considered a category 2 carcinogen, with long-term exposure increasing the cellular rate of mutagenesis [37]. However, toxic effects are highly dependent on the vanadium species being presented, as different vanadium compounds will result in different organ accumulation and residence time [1,10]. Additionally, the probability of reaching effective toxic levels through normal dietary intake is very low. Intoxication is only likely to occur from exposure to highly enriched environments, such as those that may be encountered in the metallurgy and petrol industries [2].

## 4. Vanadium Speciation in Biological Media

Upon absorption in the intestine or lungs, vanadium enters the bloodstream, where its oxidation state may be altered depending on the administered species, oxygen tension, or presence of biological reductants, such as NADH and glutathione, causing interconversion between V(V) or V(IV) forms. Within the blood plasma, vanadium predominantly binds to Serum transferrin (Tf), the systemic iron transporter [14,38,39,40]. Additionally, binding to Human Serum Albumin (HSA) and Immunoglobulins has been observed [41,42,43,44]. Vanadium ions may bind to HSA at the recognized metal ion binding sites or the reduced cysteine residue, while vanadium complexes can interact with HSA non-specifically or at the known drug binding sites [45,46]. Notably, the binding of vanadium to HSA has been reported to enhance the activity of vanadium compounds.

Mechanisms governing the cellular uptake of vanadium exhibit significant variability depending on its speciation within the extracellular environment. Most vanadium is believed to enter cells bound to Tf when vanadium-containing Tf is recognized by the Tf receptor [38]. Vanadium bound to HSA can also be taken up by cells via HSA cell surface receptors [47]. Additionally, vanadium in the blood plasma can be associated with low molecular weight compounds like phosphate, citrate, or lactate [48,49], potentially allowing entry into cells through interactions with their corresponding transporters [1]. Negatively charged vanadium oxides, including HVO_4_^2−^, might access cells through anion channels such as those used for phosphate and sulfate [50]. Moreover, certain vanadium compounds, like those linked to hydroxypyridinones, could enter cells by passive diffusion through the cell membrane [21,51].

Once inside cells, V(V) is often believed to undergo reduction, primarily by NADH or glutathione, leading to its presence mainly in the V(IV) state as the vanadyl cation. Intracellularly, most vanadium is incorporated into ferritin, the protein responsible for iron storage [40,41]. In addition, a labile and readily exchangeable fraction of intracellular vanadium is associated with phosphate and low molecular weight organic acids [41]. Furthermore, vanadium can interact with phosphate-rich molecules, including ATP and DNA [5,9]. Interestingly, when incorporated into red blood cells, vanadium extensively binds to hemoglobin [41,52].

## 5. Antidiabetic Effects of Vanadium

### 5.1. Vanadium Therapy: Studies in Animal Models

The use of vanadium in diabetes treatment has been of scientific interest since 1985 when Heyliger et al. [35] published their first study. In this research, the authors investigated the effects of sodium orthovanadate on STZ-induced diabetic rats. They demonstrated that vanadate administered in drinking water controlled the high blood glucose and prevented the decline in cardiac performance due to diabetes. This discovery triggered a great deal of work demonstrating the beneficial effects of vanadium in the treatment of diabetes and several studies were carried out using vanadate and vanadyl inorganic salts.

One significant effect of vanadate administration is a sustained decrease in blood glucose levels. This suggests that vanadate can improve glucose homeostasis in conditions where there is a lack of insulin production. Additionally, vanadate has been shown to substantially improve glucose homeostasis in hyperinsulinemia insulin-resistant animals, indicating that vanadate may be effective in improving insulin sensitivity and addressing insulin resistance, a common characteristic of T2D [35,53,54,55].

On the other hand, it has been demonstrated that vanadyl compounds can enhance the effectiveness of administered insulin. Since vanadyl sulfate was reported to be 6–10 times less toxic than vanadate, this vanadium form was extensively investigated for its insulin-like effects. Vanadyl derivatives have shown partial correction of pancreas alterations, suggesting a potential beneficial effect on the insulin-producing cells in the pancreas [56,57,58,59].

In vivo, at low doses, vanadate and vanadyl have been shown to repeatedly counteract both the hyperglycemia and hyperlipidemia of diabetes, T1D and T2D diabetic animal models. Finally, the long-term effects of vanadyl treatment on glucose homeostasis have been observed even after the cessation of treatment, indicating a sustained benefit [60].

While these findings are promising, there are some potential toxic effects associated with vanadium salts [61,62,63,64,65,66]. Experiments have been conducted to address the challenges associated with the continuous administration of vanadium compounds and the subsequent accumulation in tissues, which can lead to significant side effects. To mitigate these issues, researchers have explored the use of vanadium compounds in the form of metal ion chelates. The administration of vanadium as a coordinated complex should help to overcome gastrointestinal side effects and enhance vanadium absorption through the gut. McNeill and co-workers performed animal studies with one of the first organic vanadium complexes, bis(maltolato)oxovanadium(IV), BMOV ((**1**), Figure 3) [67,68,69]. They demonstrated that this compound effectively produced glucose-lowering effects at a significantly lower dose than previously used for inorganic vanadium salts, without any apparent toxicity. The effectiveness of bis(ethylmaltolato)oxovanadium(IV), BEOV ((**2**), Figure 3) and bis(isopropylmaltolato)oxovanadium(IV), BIOV ((**3**), Figure 3) complexes have also been reported [70,71].

These compounds exhibit potential as hypoglycemic agents, indicating their capability to reduce blood glucose levels. Moreover, they have demonstrated enhanced potency and efficacy when compared to vanadyl sulfate in glucose-lowering ability; however, this effect was not correlated with blood vanadium levels.

Other strategies to reduce vanadium toxicity included the synthesis of ligands containing a pyrone skeleton as a coordination motif and an antioxidative group derived from natural antioxidants. The antidiabetic effects of bis((5-hydroxy-4-oxo-4H-pyran-2-yl)methyl 2-hydroxybenzoatato)oxovanadium (IV) (BSOV) ((**4**), Figure 3) were evaluated using STZ-induced diabetic rats. In comparison to BMOV, used as a positive control, BSOV demonstrated remarkable results. It effectively reduced blood glucose levels, ameliorated hepatic and renal damage in diabetic rats, and improved lipid metabolism [72].

Oral administration of bis ((5-hydroxy-4-oxo-4H-pyran-2-yl)methyl benzoatato)oxovanadium (IV) (BBOV) ((**5**), Figure 3) restored the blood glucose to normal levels and ameliorated glucose tolerance in 4 weeks treatment on streptozotocin (STZ)-induced diabetic rats [73].

There has been a significant advancement in the potential application of vanadium compounds with pharmacological properties through the development of new vanadium(V) and (IV) complexes with various organic ligands. The primary objective was to improve the absorption, tissue uptake, and intracellular behavior of vanadium compounds, ultimately leading to a reduction in the required dosage for achieving optimal effects. Numerous ligands have been synthesized for coordinating with vanadyl due to its lower toxicity compared to vanadate. Additionally, vanadyl has a higher affinity for blood and cell membrane transporters, along with lower residence time in the body and increased renal clearance. Furthermore, it forms more stable bonds with organic ligands and consistently demonstrates antidiabetic effects [5].

Bis(allixinato) oxovanadium(IV) ((**6**), Figure 3) is another complex with VO(O_4_) coordination mode, which is a potent agent that has been shown to improve hyperglycemia not only in STZ mice but also in obesity-linked KKAytype-2 diabetic mice model. This complex incorporates allixin, a garlic component known for its remarkable in vitro insulin-mimetic activity, demonstrated by its ability to inhibit free fatty acid (FFA) release and enhance glucose uptake in isolated rat adipocytes. These authors believe that the lipophilicity of this vanadyl complex plays a crucial role in its insulin-mimetic properties [74,75].

Other interesting candidate complexes of vanadyl, with ligands such as hydroxypyridinone derivatives ((**7**), (**8**), and (**9**)), Figure 3), have been prepared and insulin-mimetic activities have been demonstrated. In vitro studies, using FFA release from isolated rat adipocytes show that all these complexes have an inhibitory effect on FFA release and that complex ((**7**), Figure 3) has significantly better insulin-mimetic activity than vanadyl sulfate [20,76].

In a study conducted on 7-week-old Zucker lean and Zucker fatty rats, promising findings were observed concerning the effects of VO(dmpp)_2_ ((**8**), Figure 3). The results demonstrated that VO(dmpp)_2_ shows potential in restoring normal glucose and lipid metabolism in Zucker fatty rats. These findings suggest that VO(dmpp)_2_ could be a potential therapeutic agent to address metabolic imbalances associated with Zucker fatty rats [77]. Remarkably, this restoration led to a reversal of several pathological pre-diabetic indicators in these rats. Specifically, the treatment with VO(dmpp)_2_ resulted in a significant reduction in body weight gain, subcutaneous fat thickness, high triglyceride (HTG) content, and insulin resistance. These results highlight the therapeutic potential of VO(dmpp)_2_ in addressing the metabolic disturbances associated with pre-diabetic conditions in this animal model. VO(dmpp)_2_ treatment in the T2D GK rats significantly decreases hyperglycemia and improves glucose intolerance acting on key proteins of the insulin pathway, thus confirming the anti-diabetic properties of this vanadium compound which may be a promising therapy for diabetes [78].

Some findings have emerged from research on bidentate ligand complexes of acetylacetonate, particularly VO(acac)_2_, ((**10**), Figure 3), and its 3-alkyl-acetylacetonate analogs [79,80].

VO(acac)_2_, when administered orally or injected, showed a sustained reduction in glycemic levels lasting up to 5 days in STZ-diabetic rats. This compound’s prolonged effect is ascribed to its stability and ability to interact with serum albumin, which significantly extends its presence in the bloodstream. The enhanced blood residence time of VO(acac)_2_ contributes to its long-lasting therapeutic impact, making it a promising candidate for managing diabetic conditions [81].

Vanadium dipicolinate complexes ((**11**), Figure 3) have been the subject of significant research work (reviewed in [82]). The advantage of these complexes, like hydroxypyridinones, lies in their various analogs, which offer an excellent opportunity to explore the structure-activity relationship concerning their anti-diabetic properties. Among these analogs, bis(6-methylpicolinato)oxovanadium(IV), ((**12**), Figure 3) and bis(5-iodopicolinato)oxovanadium(IV) ((**13**), Figure 3) have demonstrated enhanced in vitro insulin-mimetic activity and greater efficacy in reducing blood glucose levels in STZ-induced diabetic rats [83].

Bis(pyrrolidine-N-carbodithioato)oxovanadium(IV) [VO(pyd)_2_] complex, ((**14**), Figure 3) was found to be the most effective among the prepared complexes with the VO(S_4_) coordination mode, being dose-dependent in the in vitro model and also in treating type 1 STZ-rats by both daily intraperitoneal injections (i. p.) and oral administration [84,85,86].

Bis(1-oxy-2-pyridinethiolato)oxovanadium(IV) VO(opt)_2_ complex, ((**15**), Figure 3) with the VO(S_2_O_2)_ coordination mode exhibited strong insulin-mimetic activity in a dose-dependent manner in an in vitro system and normalized the blood glucose levels in STZ-rats when given daily injections or oral administrations [87,88].

Moreover, the VO(opt)_2_ complex was tested in ob/ob mice, an animal model for obese T2D. During a 15-day oral treatment with the complex, there was a clear dose-dependent reduction in glucose, insulin, and triglyceride levels in the bloodstream of these mice [89].

The interest in this type of complexes has increased, as shown by the growing number of publications since 2009 [90], and several complexes of vanadium with potential for the treatment of DM have been developed and tested in animal models showing similar effects, but less toxic features or non-observable adverse effects. Vanadium(IV)-diamine complex, shows hypoglycemic activity and a reduction in testicular atrophy [91]; [Bis(2,2,6,6-tetra methyl-3,5-heptanedione)(imidazol)oxovanadium(IV)], VO(BHED) ((**16**), Figure 3) reduces serum glucose levels in animals and behave as inhibitors to suppress the overexpression of PTP-1B enzyme. [Bis(2,2,6,6-tetra methyl-3,5-heptanedione)(imidazol)oxovanadium(IV)], 4-imi, ((**17**), Figure 3) reduces serum glucose levels in animals and behave as inhibitors to suppress the overexpression of PTP-1B enzyme [92]. The compound N,N’-1,3-propyl-bis(salicyladimine)]oxovanadium (IV), (BPOV) ((**18**), Figure 3) has demonstrated promising insulin-enhancing and antidiabetic properties [93].

Vanadium(IV) complexes of Schiff bases, derived from acetohydrazide, ((**19**), Figure 3), (HL1-3) or 4-aminoantipyrine (HL4-7), ((**20**) and (**21**), Figure 3), have been prepared and in vivo effects of vanadium complexes were studied using STZ-induced diabetes model in rats. Results revealed that the oral management of vanadium complexes significantly reduced the blood glucose level in rats suffering from diabetes [94].

Oxidation states V(III) and V(V) have also been explored for the insulin-mimetic properties of their complexes. An interesting study was conducted to investigate if the chemical valence and anti-oxidation effects of vanadium compounds are involved in the antidiabetic effects observed in STZ-induced diabetic rats treated with different vanadium compounds. Oral administration of various organic V(III, IV, V) compounds with dipicolinate (dipic), and (dipic-Cl), ((**22**), (**23**), (**24**), Figure 3) showed that the V(V) compound appears to be more effective than V(III) and V(IV) oxidation states, at lowering high blood glucose in STZ-induced diabetic rats, in contrast to previous studies in which the V(IV)−maltol complex, (BMOV) was the most effective [95,96].

A series of oxovanadium complexes prepared with triazole derivatives with hydroxybenzyl moieties has also shown promising insulin-like activity, reducing glycemic levels and controlling cholesterol and triglycerides in the BALB/c mice model of type 2 diabetes [97].

### 5.2. Vanadium Therapy: Studies in Humans

Human clinical studies with vanadium compounds for the management of diabetes started in the 1990s, following the promising results obtained with rodent diabetes models.

Clinical trials in humans are usually classified as Phase 1, 2, or 3. In the first type, the new drugs are administered in healthy humans to evaluate the eventual toxic effects. According to the toxicity, then the investigation can move on to Phase 2 clinical trials aiming to determine the effective dosage. In the following step, the treatment is administered to patients suffering from a specific medical condition. In the end, results are shared with the competent agencies, for approval for commercialization to humans [98].

Despite these classifications, the existent studies considering the use of vanadium to treat diabetes do not always satisfy the common requirements. Smith et al. [99] published a review paper summarizing the evaluation of the antidiabetic activity of vanadium in T2D patients in which it has been demonstrated that the relevance of the obtained results is ambiguous due to the study design. The original criteria for the review considered studies as valid when including placebo-controlled trials, an oral dose of vanadyl sulfate between 30–150 mg daily, at least two months of treatment, and with a minimum of 10 diabetic patients. Amongst the various listed investigations, only 5 works were considered valid by the authors’ criteria [58,100,101,102,103]. In general, these studies presented a small sample size and short treatment durations, and, therefore, vanadium cannot be easily recommended as antidiabetic therapy based on these studies. Nowadays, FDA (Food and Drug Administration) agency guidelines require a randomized, placebo-controlled trial with the treatment of oral vanadium compounds, considering at least 2 months and 10 diabetic patients per study [98].

Nevertheless, the first report on the use of vanadium salts for the treatment of diabetes dates from 1899 [104]. During some months, the authors first tried the administration of sodium metavanadate on themselves and after this on a group of 60 patients, including 3 diabetics. This study was considered as a “Phase 0” clinical trial due to its preliminary nature and results suggested some lowering on glucose levels without adverse effects.

Of the most systematic experiments, the study developed by Cohen and colleagues in 1995 was the [100] first clinical trial using simple inorganic vanadium compounds to treat diabetic individuals, in this case, vanadyl sulfate. The drug was orally administered (50–125 mg/day), for 2 to 4 weeks. Results have shown improved plasma glucose levels and daily insulin requirements. In T2D subjects, it was verified an increase in insulin sensitivity, and a reduction in plasma glucose levels and glycosylated hemoglobin (HbA1c). The main side effects were gastrointestinal intolerance, mainly nausea and mild diarrhea, in some of the patients. These studies were sustained for up to 2 weeks after the end of administration of the compound.

In the same year, Goldfine and co-workers also published a study in which a different inorganic vanadium compound, sodium metavanadate was orally given to insulin-dependent diabetes mellitus (IDDM) patients and non-insulin-dependent diabetes mellitus (NIDDM) in a dosage of 125 mg/day for 2 weeks. It was found that the vanadium administration conducted a decrease in cholesterol levels in both groups as well as an improvement in insulin sensitivity in NIDDM patients [105]. However, some patients experienced mild gastrointestinal symptoms as those described in the study performed by Cohen.

In 1996, a study performed by Halberstam et al. [102] at the Albert Einstein College of Medicine, inspected the effects of oral vanadyl sulfate (100 mg/day) in NIDDM patients and non-diabetic subjects, considering the administration of 2 weeks of placebo and 3 weeks of the vanadium compound. The plasma glucose remained unchanged in non-diabetic patients, and fasting plasma glucose and HbAlc decreased in NIDDM patients. Only minor gastrointestinal discomfort and stool discoloration have been reported as side effects.

In the same year, Boden and colleagues [101] designed an investigation comprising the oral administration of 50 mg of vanadyl sulfate twice daily for 4 weeks in NIDDM patients, followed by more than 4 weeks in which patients were treated with a placebo. The results evidenced the decreased fasting plasma glucose levels during vanadyl administration, as well as during the administration of a placebo. Similarly, some side effects such as diarrhea, flatulence, slight nausea, and abdominal cramps were observed.

Later in 2001, Cusi and colleagues studied the effect of vanadyl sulfate (150 mg/day) in T2D over a period of 6 weeks and the authors verified a significantly improved glycemic control, indicated by a decrease in fasting plasma glucose and HbAlc levels. The treatment was well tolerated, with minor side effects, mainly related to the gastrointestinal tract as found in the previous clinical trials [58].

In between, some other relevant studies have been reported such as the one performed by the Goldfine/Kahn team in 2000 [103]. Herein, vanadyl sulfate was orally administered for 6 weeks in T2D patients and investigations found a decrease in the fasting blood glucose as well as in HbA1c. Moreover, the treatment significantly increased some insulin-mediated activation of insulin receptors, like IRS-1 protein kinase and PI3K, without increasing insulin secretion. Once again, some gastrointestinal intolerance has been verified. The authors concluded that the treatment was apparently well tolerated but they also stated that the long-term safety of administration of this compound has not been assessed.

Another study [106] has shown that a randomized placebo-controlled clinical trial involving a total of forty subjects in which sodium monovanadate (100 mg/day) was administered to T2D patients over 6 weeks conducted to a reduction in fasting blood glucose, HbA1C, total cholesterol, and low-density lipoproteins.

Later, Jacques-Camarena and co-workers [107] investigated the effect on insulin sensitivity and results showed that the administration of vanadyl sulfate for 4 weeks (50 mg twice/day) did not modify insulin sensitivity, but increased triglyceride concentrations in obese T2D patients with impaired glucose tolerance compared with the placebo group. The undesired effects reported were nausea, abdominal pain, and diarrhea, but with low relevance since it was verified for only one patient with a previous history of intestinal disorders.

In addition to the inorganic salts of vanadium tested, organic forms comprising chelating units have also been investigated in human subjects. Of this, and as previously described for studies using rodents, the most representative family is hydroxypyridinone ligands, particularly, 3- hydroxy-4-pyridinones. In comparison with animal tests, the doses are lower, and thus slightly antidiabetic activity was observed (reviewed in [5]).

BEOV ((**2**), Figure 3) was the vanadium complex selected for the first clinical trials, which completed Phase I and then advanced to Phase II studies. This compound is structurally related to BMOV ((**1**), Figure 3) which was first reported by McNeill and Orvig and tested in animals as described above [67]. BEOV is the ethylmaltol analog of BMOV and was selected based on its better performance in the structure-activity relationship investigations carried out with a set of other maltol-derived vanadium complexes [108].

In the first set of experiments, the complex was tested in single doses (10–90 mg) orally given to 40 non-diabetic subjects, and no side effects were described. Vanadyl sulfate has been tested as a control and studies revealed that the bioavailability of vanadium from BEOV was three times higher than from the inorganic salt tested. No adverse health effects were observed, and blood parameters also remained within normal values throughout the study. Then, in Phase II trials, the safety and efficacy of 20 mg/day were evaluated for 28 days in T2D individuals, followed by 14 days without therapy. Results have shown a decrease in fasting blood glucose when compared to placebo subjects [108,109].

However, the clinical studies conducted by Akesis Pharmaceuticals Inc., finished in 2009. The company announced that upon three months of preclinical safety studies, some renal problems have been described thus compromising the use of the complex for antidiabetic purposes (reviewed in [90,110]).

A few years later, another study assessed the long-term efficacy and safety of oral vanadyl sulfate in T1D patients. Firstly 80–120 mg/day was given for 2–5 weeks and then a higher dose (225–300 mg/day) was administered for 30 months. Results have shown that the fasting blood glucose and insulin requirement of the patients was significantly reduced, without major side effects, except for some mild diarrhea episodes at the beginning of treatment. The study pointed out the effectiveness and long-term safety of vanadium administration in T1D patients [111].

In the same year, Willsky et al. [112] continued the investigations to obtain insight into vanadium pharmacokinetics and biodistribution. Vanadyl sulfate (25–100 mg/day) was orally administered for 6 weeks to T2D patients and elemental V was then determined in serum, blood, and urine. Authors concluded that vanadium pools other than total serum vanadium were probably related to its insulin-like activity thus pointing out the need for further investigations on the coordination chemistry of metabolites and interaction of proteins with vanadium chelates.

Very recently, a randomized, double-blind, placebo-controlled clinical trial was reported in which the IRS-1 regulation and the clinical responses upon the administration of vanadium-enriched yeast supplementation in 44 obese T2D patients were investigated for 12 weeks [113]. The supplementation contained vanadium pentoxide (0.9 mg/day) and the results demonstrated that their fasting blood glucose and HbA1c decreased, while their insulin sensitivity increased.

Overall, although the effects of vanadium, both considering inorganic salts or ligand-based coordination complexes, are well supported, there are relatively few studies on human patients with positive results, and they are generally short-lived. (Table 1). Therefore, vanadium administration for the treatment of human diabetes remains relatively limited, and major improvements and novel strategies must be taken into consideration to reach the desired long-term antidiabetic activity without compromising the safety of the treatment.

**Figure 4:**
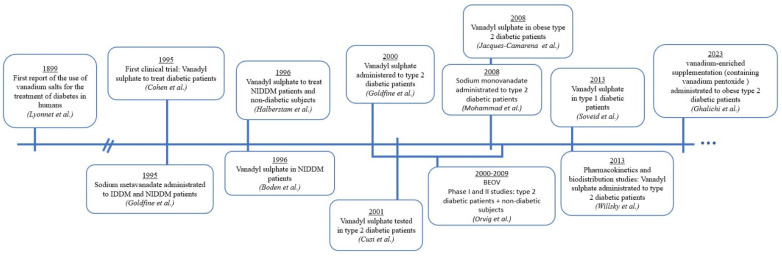
Timeline of the most relevant human studies regarding the use of vanadium compounds to treat diabetes [58,100,101,102,103,104,105,106,107,108,109,111,112,113].

An overview of the most relevant studies on humans regarding the use of vanadium compounds to treat diabetes is depicted in Figure 4. The timeline shows that despite the great findings reported, in almost 125 years it was not possible to find a lead compound with applicability in clinics. This fact is critical in a drug discovery pipeline and points out the challenges that these types of compounds offer when considered in an industrial-scale study.

## 6. Insights on Vanadium Mechanism of Action in Glucose Homeostasis

**Figure 5:**
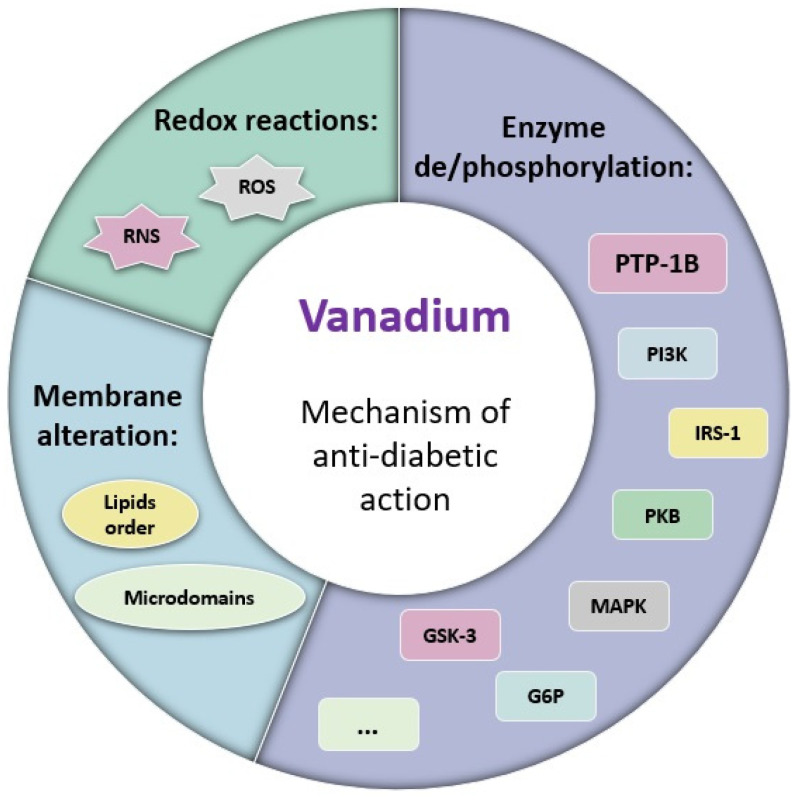
Scheme summarizing the described mechanisms of antidiabetic effect for vanadium compounds involving: (1) Enzyme de/phosphorylation; (2) Membrane alteration; and (3) Redox reactions.

In the last years, different studies have been conducted to obtain insights into the mechanism of the antidiabetic action of vanadium compounds. Several hypotheses have been formulated, comprising enzyme (de)activation, redox reactions, and membrane alterations (Figure 5). Nevertheless, the most accepted mechanism relates to the inhibition of tyrosine kinases and phosphatases [114,115,116], particularly protein tyrosine phosphatase 1B (PTP-1B) in the insulin signaling cascade. But firstly, it is important to understand the insulin signaling pathway.

Insulin is one of the most fundamental hormones as it regulates glucose homeostasis. When blood glucose levels rise after absorption of sugars through the intestinal tract, the pancreatic β-cells increase insulin secretion [117]. Then, a signaling cascade is initiated in the insulin receptors (IR) which are present in the membrane of many cells, such as hepatocytes and adipocytes. Glucose diffuses through the cell by the glucose transporter 4 (GLUT4), and an upregulation of protein synthesis and glycogenesis occurs in striated muscle cells, as well as of lipogenesis in adipocytes and hepatocytes, while a downregulation of gluconeogenesis is verified in hepatocytes [117,118].

Upon the binding of insulin to the insulin receptor (IR) alpha units, autophosphorylation of the beta-units tyrosine residues occurs, which allows the binding of the insulin receptor substrate (IRS-1) which is phosphorylated and activated.

IRS-1 then binds to the p85 subunit of phosphoinositide 3-kinase (PI3K), activating it and causing its catalytic p110 subunit to phosphorylate phosphatidylinositol biphosphate (PIP2) into triphosphate (PIP3). In turn, PIP3 activates phosphatidylinositol-dependent kinase (PDK1) which then phosphorylates protein kinase B (PKB/Akt), among others (reviewed in [119]). PKB is then central for the translocation of GLUT4 vesicles, the activation of glycogen synthase (GS), and the activation of ATP citrate lyase (fatty acid synthesis). It also activates mTORC1, promoting protein synthesis and cell growth and proliferation, and activates SIK2, inhibiting gluconeogenesis. Afterward, IR is dephosphorylated by PTP-1B at the beta subunits of tyrosine residues and this event blocks IRS-1 binding and interrupts the signaling cascade. When insulin concentration is low, the auto-phosphorylation rate of IR drops, while PTP-1B activity is not directly affected by insulin. IR signaling is, in this way, dynamically regulated (reviewed in [118]). The activity of PTP-1B is one of the main negative regulators of IR signaling, decreasing its phosphorylation, and the over-expression of PTP-1B has been related to the development of insulin resistance. Therefore, the use of PTP-1B inhibitors holds the potential to improve the sensitivity of the insulin receptor and ameliorate insulin resistance [120].

Many investigations regarding vanadium antidiabetic activity support the hypothesis that the already mentioned inhibition of protein tyrosine phosphatases by vanadium compounds is due to the vanadate-phosphate analogy. The structural similarity of vanadate to phosphate [121] allows its binding to tyrosine residues of PTP-1B; however, this binding is more stable than normal phosphorylation, irreversibly deactivating PTP-1B. Since vanadium suppresses the dephosphorylation of tyrosine residues of the β-subunit of insulin receptors [122], the IR, therefore, stays phosphorylated even when insulin levels decrease, and the signaling cascade is maintained, increasing sensitivity to insulin.

For this reason, some authors argue that vanadium is not so much an insulin-mimetic, but more a signal modulator or insulin enhancer, for without the simultaneous activation of the IR by insulin the signal transduction would be insufficient.

The inhibition of PTP-1B by vanadium results also in the phosphorylation of IRS-1, leading to the activation of PI3K [90,123], which in turn increases the number of GLUT4 transporters and thus their translocation [124]. This pathway was confirmed for example for BMOV [125] and VO(dmpp)_2_ [78] in which VO complexes inhibit PTP-1B and activate phosphatidylinositol3-kinase/Akt signaling by stimulating tyrosine phosphorylation of IR and IRS-1 (reviewed in [5,119]).

Srivastava et al. [126] reviewed the mode of action of BMOV and emphasized the participation of this complex in the induction of phosphorylation of PKB, glycogen synthase kinase-3 (GSK-3), and forkhead box protein 1 (FOXO1) thus contributing to the glucoregulatory responses. Due to the activation of the PI3K pathway, the PKB is phosphorylated, and downstream targets are ultimately activated leading to the regulation of glucose transport gluconeogenesis, and glycogen synthesis.

Of note, in the past some studies mentioned that the antidiabetic effects of vanadate are independent of the IR and IRS-1 phosphorylation [127] but later, further investigations demonstrated that vanadium compounds trigger insulin signaling, involving amongst others, the activation of IRS-1 [128].

Another study suggests that vanadium can also inhibit PTP-1B, thus increasing the activity of insulin-like growth factors, and therefore stimulating the production of GLUT4 transporters [129], increasing the biosynthesis of glycogen [130], and decreasing gluconeogenesis, through the blocking of phosphoenol pyruvate carboxykinase (PEPCK) [131] and G6P [132], and inhibition of lipolytic pathways [133].

Recently, a clinical trial in obese T2D patients has shown that vanadium pentoxide allowed the regulation of different insulin signaling cascade players, particularly PTP-1B, mitogen-activated protein kinase (MAPK), and nuclear factor kappa B (NFƘB) gene expression levels [113].

It is reported that vanadium can activate PKB (reviewed in [90]) but in opposition, it was also reported that vanadium can inhibit different enzymes, such as phosphodiesterases [134,135] and phosphoglucomutase [116,136]. As an example, it was found that vanadium impacts insulin resistance and improves glucose uptake by altering the nitric oxide (NO)/cGMP/protein kinase (PKG) signaling pathway through the inhibition of phosphodiesterases [134].

Vanadium has also been shown to deactivate various other phosphatases by coordination with their active centers, as for SHP-1, SHP-2, and the PTP associated with insulin-like growth factor receptor (IGF-IR), which may potentiate its antidiabetic effect but also causes concerns regarding its specificity of action [6]. Also, it has been described that vanadium can activate glucose-6-phosphate dehydrogenase in mammalian cells as well as vanadate compounds activate the tyrosine kinases p56Ick and p59fyn [137,138].

Another proposed mechanism for the antidiabetic action of vanadium is based on the eventual ROS and RNS (reactive oxygen and nitrogen species, respectively) produced in V metabolism [139,140,141]. The free radicals produced can also inhibit PTP-1B by oxidatively targeting the Cys residue present in this protein (reviewed in [142]). Crans and colleagues pointed out the relevance of coordination chemistry and redox chemistry, particularly the vanadium oxidation state (3, 4, or 5) in different vanadium complexes on their antidiabetic activity [82]. It has also been reported that some vanadium forms can bind to the oxygen atoms of the Tyr side groups thus leading to redox reactions, therefore probably modifying some proteins in the insulin signaling cascade, namely PTP-1B through these redox processes [143].

In addition, Crans and colleagues found that the interaction of vanadium with cell membranes results in the stabilization of vanadium complexes and conduces to alterations in membrane proteins that may be relevant for the anti-diabetic effect, impacting the uptake and action of the vanadium compounds [51,144]. Particularly, the authors have shown that BMOV decreases lipid order while increasing the association of IR in specialized nanoscale membrane microdomains. It was suggested that the observed antidiabetic effect may be mainly caused by these modifications in the lipid order of the cell surface rather than due to the direct interaction of vanadium with the IR [145].

Overall, these findings pointed out that vanadium can participate in numerous biological processes, particularly by interacting with several membrane and cytosolic proteins, which may be relevant for its both beneficial and potential adverse effects [6]. The most relevant mechanisms of action attributed to vanadium antidiabetic activity are summarized in Figure 5.

Since it is well documented the activity of vanadium on PTP-1B inhibition, this interaction may be considered a promising target for antidiabetic drug discovery. Moreover, it is described that PTP-1B is overexpressed in diabetic and obese patients, suggesting the interesting use of inhibitors in the treatment of diabetes and obesity. Despite the well-demonstrated effect of many PTP-1B inhibitors, particularly for vanadium, there is no clinically used drug for this purpose, which represents a great opportunity for vanadium compounds in the treatment of such metabolic diseases [146].

## 7. Conclusions and Future Perspectives

Clinical trials with vanadium compounds have encountered significant challenges and limitations. Despite promising results, these trials do not comply with current FDA regulations. The studies often had outdated designs, and most included a limited number of subjects or were carried out in a short time. Additionally, the formulation of these compounds as potential drugs received inadequate attention, leading to concerns about the low bioavailability presented in those studies.

A consensus among researchers is that increasing the bioavailability of these compounds could significantly strengthen their effectiveness. Reinvestigation of vanadium compounds would presumably require some improvements in compound design or delivery systems to enhance its efficacy. And, with our current knowledge of vanadium biochemistry, it is most likely that different forms of vanadium and vanadium compound formulations would be chosen for human studies.

Additionally, vanadium’s known toxicity, especially in certain forms, must be considered and further research is needed to better understand its safety profile and optimal therapeutic use. While toxicity from normal dietary intake is minimal, it is regarded as a hazard in highly enriched environments, such as metallurgy industrial settings.

The mechanism of the antidiabetic action of vanadium compounds is a complex and multifaceted process involving several pathways in insulin signaling and glucose homeostasis regulation, that is not fully understood. One of the most accepted mechanisms of vanadium’s antidiabetic action centers around the inhibition of protein tyrosine phosphatase 1B (PTP-1B), a critical regulator in the insulin signaling cascade. Despite these promising insights into vanadium’s potential as an antidiabetic agent and its targeting of PTP-1B inhibition, there is currently no clinically used drug employing vanadium for this purpose. Additionally, vanadium’s interaction with cell membranes and membrane proteins may alter lipid order and affect the organization of insulin receptors in specialized membrane microdomains, potentially playing a role in its antidiabetic effects.

In conclusion, the search for alternative treatments and therapeutic adjuvants remains critical to improving the management of diabetes and reducing its impact on global health. From our perspective, deep insights into the pharmacological effects of vanadium compounds remain incompletely understood. Consequently, we maintain the view that there is still a substantial amount of research to be carried out in this area. The multifaceted mechanisms of action of vanadium compounds present a rich area of research and development in the pursuit of effective treatments for diabetes and obesity. More studies and clinical trials are needed to fully harness the potential of vanadium compounds in treating these metabolic disorders.

## Figures and Tables

**Table 1:** Summary of the experimental details and main results obtained in the most relevant clinical trials regarding vanadium antidiabetic activity.

Oral Treatment	Experimental Design	Main Results	Reference
Vanadyl sulfate 100 mg/day (50 mg twice daily) 3 weeks active 2 weeks follow-up	Single-blind, placebo-controlled 6 T2D patients No diabetic subjects	Reduction in plasma glucose and HbA1c Reduction of FFA Gastrointestinal intolerance	[92]
Sodium metavanadate 125 mg/day (50 + 50 + 25 mg/daily) 2 weeks active 2 weeks follow-up	Non-randomized, non-placebo-controlled 5 T1D patients 5 T2D patients No diabetic subjects	No changes in insulin sensitivity in T1D patients No changes in plasma glucose and HbA1c in both diabetic types Decrease of total cholesterol in both diabetic types Mild gastrointestinal side effects in both diabetic types	[97]
Vanadyl sulfate 100 mg/day (50 mg twice daily) 4 weeks active 4 weeks follow-up	Single-blind, placebo-controlled 8 T2D patients No diabetic subjects	Reduction in plasma glucose No information about HbA1c Gastrointestinal side-effects	[93]
Vanadyl sulfate 100 mg/day (50 mg twice daily) 3 weeks active No follow-up	Single-blind, placebo-controlled 7 T2D patients 6 non-diabetic subjects	Reduction in plasma glucose and HbA1c Decrease of total cholesterol Reduction of FFA Minor gastrointestinal side-effects Stool discoloration	[94]
Vanadyl sulfate 75, 150, and 300 mg/day (25, 50, and 100 mg/3 times daily) 6 weeks active 2 weeks follow-up	Single-blind, placebo-controlled 16 T2D patients No diabetic subjects	Reduction in plasma glucose and HbA1c Some gastrointestinal intolerance	[95]
Vanadyl sulfate 150 mg/day (50 mg 3×/daily) 6 weeks active 6 weeks follow-up	Single-blind, non-placebo-controlled 11 T2D patients 5 non-diabetic subjects	Reduction in plasma glucose and HbA1c Minor gastrointestinal side-effects	[51]
BEOV Phase I—10–90 mg/day 2 weeks active No-follow up Phase II—20 mg/day 28 days 14 days follow-up	Single-blind, placebo-controlled 40 non-diabetic subjects Single-blind, placebo-controlled 7 T2D patients	No information about plasma glucose and HbA1c No side effects Reduction in plasma glucose and HbA1c Renal side effects	[101]
